# Hemopoiesis in the Thymus

**DOI:** 10.1155/1995/69454

**Published:** 1995

**Authors:** Marion D. Kendall

**Affiliations:** 1 Thymus Laboratory The Babraham Institute Cambridge CB2 4AT United Kingdom; 2 Pharmacology Department UMDS London SE1 7EH United Kingdom

**Keywords:** B cells, erythrocytes, granulocytes, hemopoiesis, mast cells, thymus

## Abstract

The presence in the thymus of hemopoietic cells other than thymocytes has been known for many years, but the extent of the hemopoietic activity of the thymus and the possible functional implications have only recently begun to receive much attention. This review summarizes the literature in this field, especially in the light of current cytokine and thymic-factor knowledge, and includes clinical relevance where possible.


*Developmental Immunology*, 1995, Vol 4, pp. 157–168
Reprints available directly from the publisher
Photocopying permitted by license only

© 1995 Harwood Academic Publishers GmbH
Printed in Singapore

### Hemopoiesis in the Thymus

MARION D. KENDALL

*Thymus Laboratory, The Babraham Institute, Cambridge CB2 4AT, and Pharmacology Department, UMDS, London SE1 7EH, UK*

The presence in the thymus of hemopoietic cells other than thymocytes has been known for many years, but the extent of the hemopoietic activity of the thymus and the possible functional implications have only recently begun to receive much attention. This review summarizes the literature in this field, especially in the light of current cytokine and thymic-factor knowledge, and includes clinical relevance where possible.

KEYWORDS: B cells, erythrocytes, granulocytes, hemopoiesis, mast cells, thymus.

### INTRODUCTION

The thymus is the major site for T-cell development, but that it may act as a site for other forms of hemopoiesis in all vertebrates, although regularly documented over the years, has been largely ignored. With the growth of good lineage markers for blood cells, it has been found that the thymus contains a range of different hemopoietic cells, often proliferating or developing *in situ*. In some cases, this has clinical significance, but in others, it may appear almost accidental or only manifest itself in abnormal situations. In any case, the occurrence of non-T-cell hemopoiesis can be used to explore the microenvironment and to give an indication of the interactive function of factors such as cytokines. This may benefit the clinical management of certain diseases and give hope for the manipulation of immune responses. This review therefore considers factors that may predispose toward the development of various hemopoietic cells in the thymus, and then discusses the presence and development of B, plasma, erythroid, and mast cells, eosinophils and neutrophils. Other hemopoietic cells are not included here, although they could also develop in the thymus. This is particularly true for natural killer and lymphokine-activated killer cells, but there is very little information about other cells, such as basophils, in the thymus.

Of critical importance in thymic hemopoiesis is the presence and potential of primitive stem/precursor/progenitor cells within the thymus and the local action of certain cytokines. In addition to the problems of finding adequate lineage markers for primitive cells, it is clear that different studies highlight, sometimes contradictory, findings. This may be a reflection of thymic embryological development. Hemopoiesis first occurs in the yolk sac, and cells from this extra-embryonic site migrate to the liver. Shortly after this, hemopoietic cells appear in the developing thymus, in some long bones (e.g., clavicle) and elsewhere (e.g., kidney). The thymus in the human embryo is hemopoietic (mainly lymphoid) by about 8 weeks, and is the most important hemopoietic organ throughout gestation (Kelemen et al., 1979). Full bone marrow development in the long bones occurs much later, and in the adult mouse, 1 in 10,000 bone marrow cells have been described as thymic colony forming units (Spangrude and Scollay, 1990). Recent research on the capabilities of stem cells populating the early mouse embryo has shown that the thymus rudiment is seeded by multipotential precursor cells that are not immediately committed to T-cell development in the thymic cellular environment (Peault et al., 1994). It is therefore quite possible that T-lymphocyte precursors in the embryo and in the adult differ in their capabilities, so that a concept of multipotential and nonlineage-restricted precursors in the embryonic thymus, and primarily T-lineage-restricted precursors in the adult is quite plausible. Indeed, Spangrude et al. (1988) demonstrated that the adult mouse has two lineages of two similar cells but $Sca^-$ also had CFU-S (colony forming units—spleen) capabilities and were probably on the erythroid line of development. Alternatively, truly multipotential stem cells of the embryo might remain in the thymus in adult life, and on occasion be

"awakened". Indeed, variations on the extent to which this might happen could be the key to many of the observations quoted in this review.

Of considerable potential for elucidating stem-cell biology is the identification of the ligand for the *c-kit* tyrosine kinase receptor (Huang et al., 1990). This is a glycoprotein that was previously called the mast-cell growth factor (MGF), the stem-cell factor (Zsebo et al., 1990), and the steel factor (Matsui et al., 1991). Stem-cell factor (SCF, a form of the *c-kit* ligand) alone or in combination with various factors (the interleukins IL-3, IL-6, IL-11, erythropoietin, and granulocyte colony-stimulating factor, or G-CSF) stimulates myelopoiesis, including erythropoiesis, as well as T- and B-cell development. It appears to be produced by a variety of cells, and one form (KL-1) is especially associated with fibroblasts, brain, and thymus (Huang et al., 1992).

Another facet of the thymus is that there may be major microenvironment differences between major lobes (left and right thymuses), as seen in repopulation studies (Ezine et al., 1984) or diseases such as myasthenia gravis (MG), as well as variations between lobes of either the left or right thymus. Furthermore, because cytokines largely shape the microenvironment, small areas within the thymus may change rapidly from day to day, or more slowly with age and physiological rhythms, under stress, disease, or as a result of the administration of drugs or toxic compounds. Thus, alterations in their levels could have profound effects on hemopoietic-lineage development.

A cytokine of major importance in hemopoiesis is IL-3. It is a multipotential cytokine stimulating the proliferation and differentiation of pluripotent hemopoietic stem cells and lineage-committed precursors of granulocytes, macrophages, eosinophils, erythrocytes, megakaryocytes, mast cells, and lymphoid cells. It is a factor for mast cell growth, is a multicolony stimulating factor, has burst-promoting activity, and is Thy1-inducing. It is produced mainly by activated T-cells and mast cells, but, of importance here, also by thymic epithelium. Its actions often need synergy from other factors (IL-1, IL-6, IL-11, *c-kit* ligand, G-CSF-1).

IL-4, another activated T-cell factor, was primarily found to act on B-cell differentiation (BCDF) and growth factor (BCGF1). However, it also acts on other hemopoietic cells such as T-, mast, and monocyte-lineage cells. In synergy with other cytokines such as IL-11, it enhances the proliferation of primitive and committed progenitors. The IL-4R molecule occurs widely on cortical epithelial cells (and some thymocytes) in the human thymus, so this cytokine could be involved in regulation of hemopoiesis within the thymus.

The T-cell-derived, B-cell growth factor for murine cells, IL-5, also has the ability to act as an eosinophil differentiating factor, albeit a late-acting cytokine. Whether this cytokine has been found to act within the thymus is not clear. In humans, some of the B-cell differentiation effects are actually carried out by IL-6. This factor has colony-stimulating actions on hemopoietic stem cells, and is known to act on thymocytes.

Even when the action of single cytokines has been defined, other roles emerge and they all interact to synergize and suppress other hemopoietic factors. IL-7, which was originally implicated in B-cell development, has been shown to be expressed in the thymus by cortical epithelial cells from fetal and neonatal thymuses (Moore et al., 1993). IL-7 and SCF are involved in T-cell development although they cannot support differentiation. GM-CSF, the granulocyte/macrophage-CSF, also causes proliferation of erythroid, dendritic, and megakaryocyte precursors. Both GM-CSF and IL-1$\alpha$ are poorly expressed by cortical epithelial cells. When cloned human thymic epithelial cells were challenged with IL-1$\alpha$, the production of GM-CSF, granulocyte-CSF, and other cytokines was strongly upregulated (Galy and Spitz, 1991). It is not known if granulocyte-CSF (G-CSF) is produced in the thymus, as it is mainly produced by activated macrophages. With stem-cell factor, it may cause proliferation of early stem cells as well as primarily acting to stimulate neutrophil colony formation from bone marrow derived cells. Finally, it has been recently suggested that CD69, which is expressed transiently in recently activated lymphocytes, may also be a marker of positively selected thymocytes, and has now been found on many hemopoietic cells. Cross-linking CD69 results in an intracellular signal in all systems investigated, so Testi et al. (1994) have suggested that CD69 molecules could act as common triggers for a variety of hemopoietic cells at different stages of their development, and therefore be of wide biological significance.

### B CELLS AND PLASMA CELLS

Thymic tissue has been used by many researchers as a source of mast-cell precursors (Ginsburg and

Sachs, 1963; Ishizaka et al., 1976), and the presence of immature cells within the gland is indicative of intrathymic development (Kendall and Warley, 1986). Both B- and plasma cells occur in the normal and diseased thymus. B-cells have a high rate of proliferation there (Pabst et al., 1989), and are generally concentrated in the medulla (Isaacson et al., 1987), and at the cortico-medullary junction, whereas plasma cells are found predominantly in the cortex (Clarke and Kendall, 1989; Abou-Rabia and Kendall, 1994). Perivascular spaces may contain both B-cells and plasma cells. Initially, B-cells were thought to be casual components of the thymus, but then as their functions became clearer, other possibilities arose. Might their presence indicate that immune responses could be enacted within the organ? Some evidence for this exists. Benner et al. (1974) presented evidence for thymic participation in the immune response (antibody formation, evidence of plasma cells and phagocytosis) after antigen dosing. In these cases, would T-cell development be affected? On the other hand, perhaps B-cells are necessary for T-cell development and their antigen-presenting capacity might influence repertoire selection. Thymic plasma cells are also a mystery. Benner et al. (1974) also showed antibody-plaque-forming cell activity in the thymus after antigen challenge, so the plasma cells probably secrete antibody. But is this a normal part of thymic activity, or does this only occur in disease?

B-cell activity in the immune response is generally associated with germinal centers in lymph nodes, so the thymus has been extensively studied in this respect. It is often assumed that thymic germinal centers are associated with autoimmune conditions, especially MG and systemic lupus erythematosus (SLE). However, germinal centers also occur in the thymus under normal conditions (Middleton, 1967; Hofmann et al., 1988; Wirt et al., 1988; Kupper et al., 1989) as well as in disease (Vetters and Barclay,1973; Rosai and Levine, 1976; Vincent et al., 1978; Fujii et al., 1983; Williams and Lennon, 1986). Furthermore, their frequency of appearance can be similar: Middleton (1967) recorded germinal centers in the thymus of 71.7% of adults 0–39 years old who died accidentally—a level of incidence comparable to that of MG patients.

A detailed immunocytochemical investigation (Wirt et al., 1988) of B-cells in nonimmunological disease (19 cases and 1 MG patient) found 25% of the patients' thymuses had active B-cell zones in medullary septa arranged as follicles. Each had an outer mantle showing IgD positivity and an inner germinal center containing dendritic reticular cells. The follicles expressed IgG, IgM, kappa, and lambda in a lacy interstitial pattern. The T-cells in the follicles were predominantly Leu3$^+$ helper/inducer cells and some were Leu7$^+$ cells (a T-cell subset and/or NK cells). All 20 thymuses examined showed a substantial minority of scattered B-cells in the septa and medulla that had the phenotype of circulating blood B-cells. This suggests that trafficking of B-cells into the medulla is common. Abou-Rabia and Kendall (1994) document the passage of cells through medullary endothelia in hypothyroidism.

The thymuses in certain models for autoimmune diseases may carry very high levels of B-cells. The mouse model for SLE has B-cells in the thymus long before the clinical demonstration of disease (Farinas et al., 1990). However, in AKR mice (prone to retrovirus associated lymphomas), an increased presence of peripheral T- and B-cells in hyperplastic and preneoplastic thymuses was postulated to be associated with a response to local antigenic stimulation (Michie and Rouse, 1991). Cells bearing the MEL-14 (L-selectin) homing receptor (the majority of B-cells in hyperplastic thymuses) were predominantly located around follicles in the enlarged medulla.

MG patients generally have hyperactive thymus glands with many showing germinal centers. Their B-cells (CD19$^+$, CD21$^+$, and IgD$^+$ or IgM$^+$) are activated (Leprince et al., 1990), produce anti-AChR antibodies *in vitro*, and their levels correlate with sera autoantibody titer and abnormal thymic histology (Safar et al., 1987). In patients with rheumatoid arthritis, peripheral CD5$^+$ B-cells secrete rheumatoid factor.

A more recent study on the properties of human thymic B-cells (Spencer et al., 1992) gives a higher representation of B-cells (CD20$^+$) in sections of thymic medulla (33 ± 4.8%) than the Wirt study (which was not quantified), and also shows them to be activated B-cells (CD19$^+$, CD20$^+$, CD22$^+$, CD35$^-$, CDw32$^-$) with about 10% expressing an indicator of cellular division (Ki67$^+$). The medullary B-cells often formed rosettes with thymocytes supporting the hypothesis that thymic B-cells are presenting autoantigens to thymocytes as part of the negative selection process. This seems plausible following murine studies of negative selection with the Vβ chain-bearing T-cells and Mls-determinant or class II I-E molecule expression. B-cells (but not macrophages, dendritic cells, or T-cells) have the M1s locus and are found early in ontogeny in the thymus, probably before B-cell development in the liver (Nango et al., 1991). Both B-cells and dendritic cells are required *in vitro* for clonal deletion in mice (Mazda et al., 1991). *In vivo*, however, thymic B-cells alone delete Mls-reactive T-cells, and induce tolerance with injected dendritic cells, while dendritic cells alone energized Vβ6 cells (Inaba et al., 1991), although the processes may be more complicated. It is now emerging that, at least in mice, the role of mature B-cells in controlling Vβ deletion is variable. Vβ11$^+$ cells require B-cell contact for deletion unlike Vβ3$^+$ and Vβ5$^+$ cells (Frey et al., 1992). Thus, B-cells are important for shaping the repertoire of T-cells to both internal and external antigens, and in establishing tolerance (Zoller, 1990).

A further complication in evaluating B-cell function is that in humans and mice, more than one type of B-cell exists. Human thymic medullary B-cells are heterogenous and may bear the CD76 antigen that appears late in maturation. They also differ phenotypically from follicle mantle and germinal center cells (Fend et al., 1991). In the mouse, thymic B-cells (CD5$^+$) differ from peritoneal cavity B-cells in several respects, especially in their inability to spontaneously produce autoantibodies (Than et al., 1992). Mouse thymic B-cells can be stimulated with MHC class II-restricted CD4$^+$ blasts and they then secrete IgM (Inaba et al., 1990), but they fail to respond to LPS or to anti-IgM plus IL-4. The cytokines IL-4 and IL-7 have both been shown to play a part in B-cell isotype switching and Ig production *in vitro* (Vandekerchhove et al., 1993). B-cells may also differ in their origins as thymic, but not peritoneal cavity B-cells can be reconstituted after irradiation and bone marrow transplantation. In this respect, thymic B-cells resemble conventional B-cells. Also fetal and adult hemopoietic stem cells have been shown to differ in their potential (Ikuta et al., 1990), so it has been proposed (Than et al., 1992) that fetal stem cells can give rise to conventional B-cells that become thymic or peritoneal cavity B-cells, but adult hemopoietic stem cells cannot form peritoneal cavity B-cells.

Whereas the preceeding studies indicate but do not prove that thymic B-cells are developed within the thymus, low frequencies of B-lineage cells (susceptible to viral transformation) are present from days 13–14 of gestation in mice. Whether these have immigrated as multipotential stem cells or as committed B-lineage cells is not known. Their frequency rapidly increases to at least 1 in 500 cells at days 15–16, which is an order of magnitude higher than similar cells in fetal liver or adult bone marrow (Kimoto et al., 1989). Mouse thymic B-cells (which are CD5$^+$, unlike human thymic, and other mouse lymphoid organ B-cells) are the first B-cells after birth to secrete IgG (Andreu-Sanchez et al., 1990), and a B-lineage transformation-associated antigen (6C3) appears on murine cortical epithelial cells 1–2 weeks after birth (Adkins et al., 1988). This antigen is also found in kidney and intestine, but in the lymphoid system, only in thymus and bone marrow, where it supports pre-B-cell proliferation and differentiation *in vitro* (Whitlock et al., 1987).

The capacity of the thymic stroma to allow B-cell development (as seen morphologically) is supported by the findings that neonatal thymectomy causes a sudden disappearance of circulating B-cells (Sprent, 1973), and reconstitution with mature T-cells partially rectifies the B-cell deficiency in X-linked immune deficient (*xid*) mice (Sprent and Bruce, 1984). Further work with *xid* mice (Karagogeos and Wortis, 1987) showed that maturation of *xid* B-cells past the pro-B or early pre-B cell is T-cell-dependent, so the necessary factors could be within the thymus.

### ERYTHROPOIESIS

Several early morphological studies refer to the presence of immature red cells within the thymus, and their occurrence was convincingly documented by Albert et al. (1965a, 1965b, 1966) in mice and man. Generally, fetal, pediatric (Taylor and Skinner, 1976), and lower vertebrate (Kendall, 1980a) thymus glands exhibit most erythropoiesis, and the levels in adult man may be low (Kendall and Singh, 1980). However, because the morphology of early erythroid cells is very similar to lymphoid cells, it is only when specific techniques for identifying erythroid cells are employed that the extent of erythropoiesis may be recognized (Kendall, 1975; Borgeois et al., 1981; Kendall et al., 1985).

In several wild bird species, thymic erythropoiesis is a regular occurrence, especially during breeding and moult (Kendall and Ward, 1974; Bacchus and Kendall, 1975; Kendall, 1975a, 1975b, 1979; Ward and Kendall 1975; Kendall and Frazier, 1979; Fronfria et al., 1985). It has been most extensively studied in the red-billed quelea, *Quelea quelea* (Fig. 1). The young after hatching (Fig. 1a) have very enlarged thymic lobes (birds usually have two

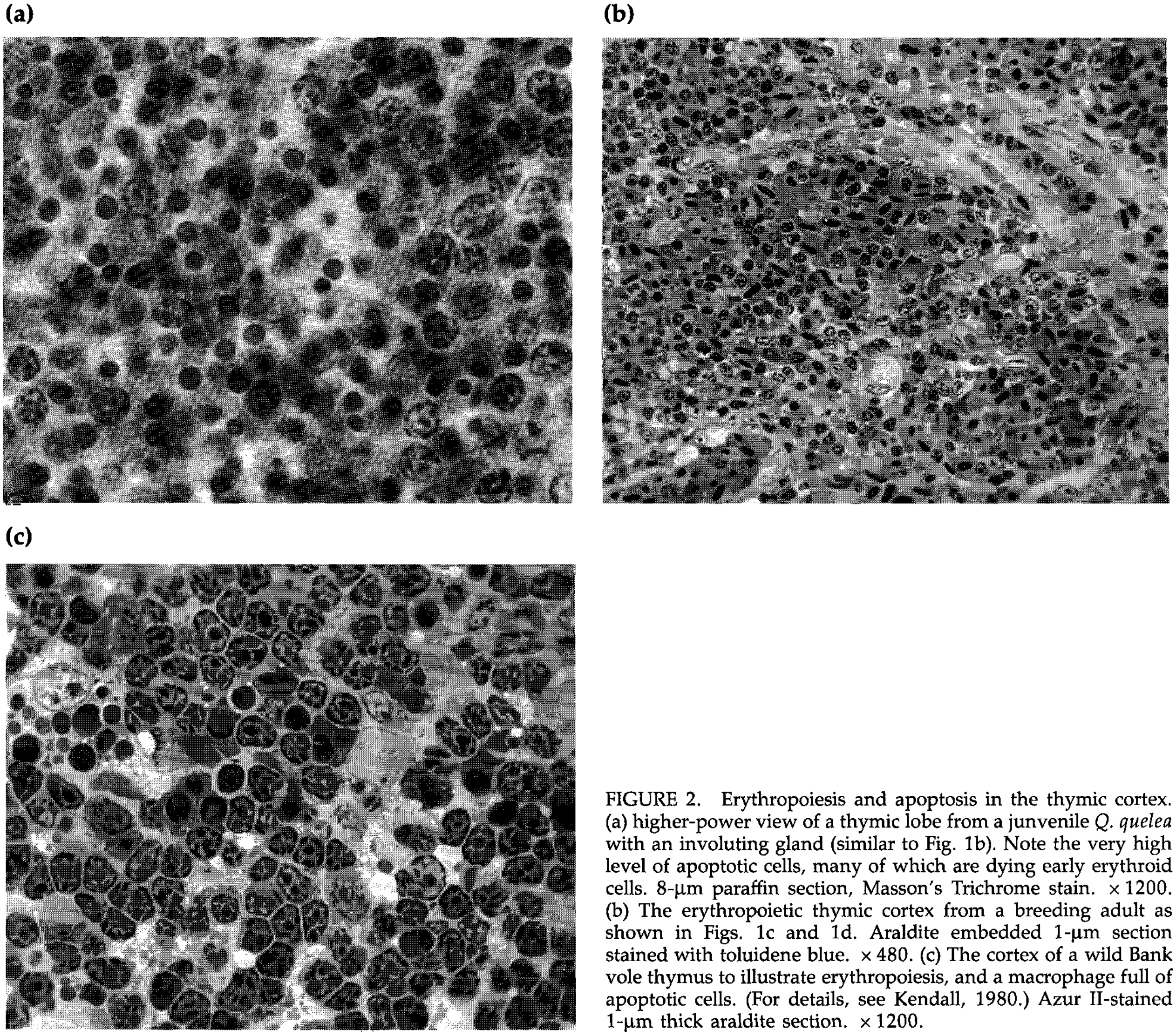

FIGURE 2. Erythropoiesis and apoptosis in the thymic cortex. (a) higher-power view of a thymic lobe from a junvenile *Q. quelea* with an involuting gland (similar to Fig. 1b). Note the very high level of apoptotic cells, many of which are dying early erythroid cells. 8-μm paraffin section, Masson's Trichrome stain. ×1200. (b) The erythropoietic thymic cortex from a breeding adult as shown in Figs. 1c and 1d. Araldite embedded 1-μm section stained with toluidene blue. ×480. (c) The cortex of a wild Bank vole thymus to illustrate erythropoiesis, and a macrophage full of apoptotic cells. (For details, see Kendall, 1980.) Azur II-stained 1-μm thick araldite section. ×1200.

Anon., 1959; Jacobs et al., 1959; Parry et al., 1959; Krantz, 1990). The relationship, if any, between removal of the thymus and the restoration of normal erythroid levels in these rare diseases is unclear, but the recognition that thymocytes can enhance or suppress erythroid colony growth may be relevant (Sharkis et al., 1986).

### MAST CELLS

Mast cells in most species display marked functional and morphological heterogeneity. The characteristics of different mast cell types has been mainly explored in rodent models and mast cell cultures. This has led to a recognition of bone marrow derived cultured mast cells (BMCMCs) from rat hemopoietic tissues (including the thymus), connective-tissue mast cells (CTMCs), and mucosal mast cells (MMCs). All of these are considered to be the progeny of multipotential stem cells (Kitamura et al., 1981). How separate the different types are, is not clear because BMCMCs have been shown to differentiate into CTMC-like cells (Nakano et al., 1985), and peritoneal CTMCs under certain culture conditions become MMC-like. Upon transfer to mast cell-deficient mice, these MMC-like cells become CTMCs. Thus, the microenvironment is very important in the development of mast cell heterogeneity.

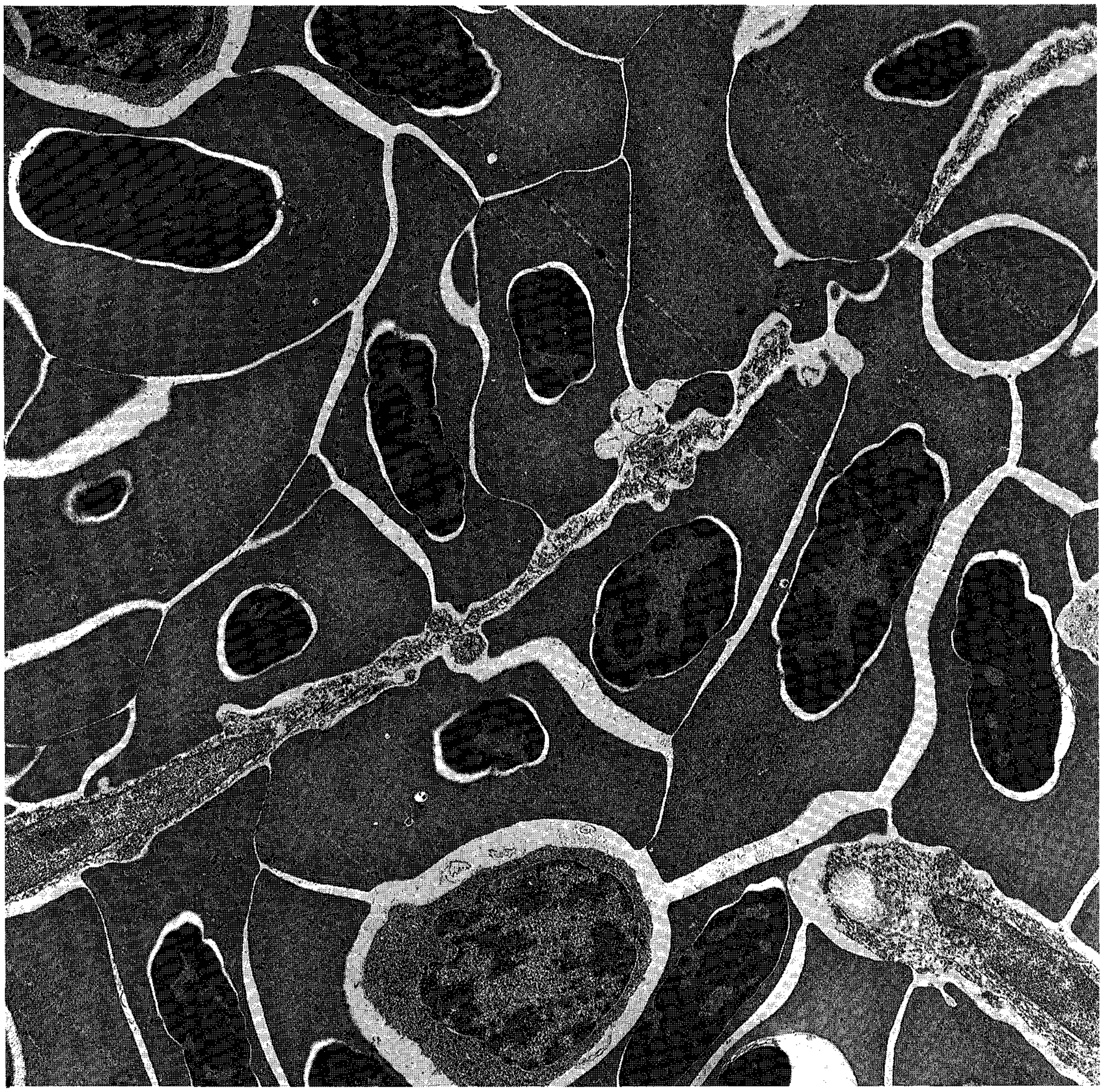

FIGURE 3. Electron micrograph of a sparrow thymus (*Passer domesticus*) showing an area of cortex almost entirely filled with erythrocytes. There are no thymocytes in this region, epithelial cells remain, and occasional large mononuclear cells are also present. Other nearby erythropoietic regions contained eosinophils and heterophils. (See Kendall, 1979.) × 8100.

Mast cells are commonly found in the capsule around the thymus and along the connective tissue septa within the gland (Frazier, 1973; Kendall and Warley, 1986; Wight, 1970). Mast cells actually within the stromal compartment (Figs. 4a and 4b) are less common except in some unusual cases, e.g., NZB mice where enormous numbers of mast cells proliferate in the cortex (Burnet, 1965), in the medulla in dystrophic chicken (Befus et al., 1981), and in induced anemia (Kendall and Blackett, 1983). Further evidence for the presence of mast cell precursors in the thymus comes from several studies where thymic tissues have been used as a source for the *in vitro* development of mast cells (Ginsburg and Sachs, 1963; Ishizaka et al., 1976), and an estimate of 17 mast cell precursors/$10^6$ thymic cells has been suggested (Kawashini et al., 1986). In other studies, X-ray microanalysis has been used to study thymic mast cell granules and the variation in potassium and sulfur content suggests the presence of immature cells in rat thymus (Kendall and Warley, 1986). Birds with the hereditary condition of muscular

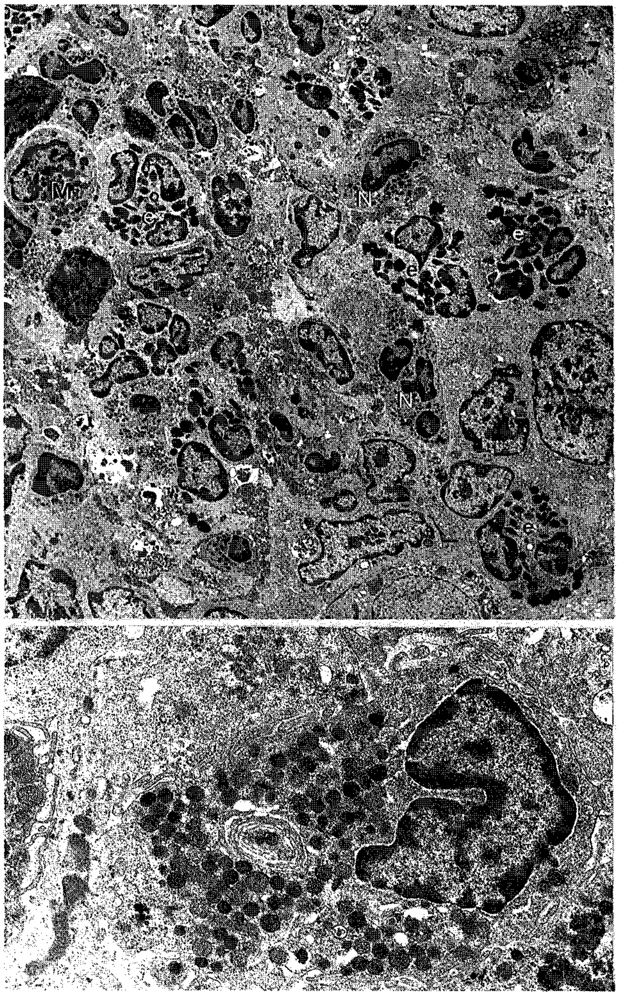

FIGURE 4. Hemopoiesis in the cortex of the thymus in a bank vole after induced anemia (reproduced with permission from Kendall and Blackett, 1983). (a) An immature mast cell (M, top left), numerous multilobed eosinophils (e), an immature neutrophil (N, top, off center), and a more mature multilobed neutrophil (N, center right). ×3000. (b) An immature mast cells illustrating the formation of the characteristic granules. ×12,600.

dystrophy have thymic abnormalities including deficiencies in thymic mast cell numbers and histamine content.

Studies with athymic and thymic rats suggest that the thymus may regulate mast cells by an inhibitory factor acting on the bone-marrow stem cell or recirculating precursor pool (Aldenborg and Enerback, 1985).

Because parasite infestations often cause massive mast cell development, the factors involved in mast cell differentiation have been widely studied. Of particular importance is that there is no MMC proliferation in T-depleted mice and rats (Ruitenberg and Elgesma, 1976; Mayrhofer and Fisher, 1979), indicating T-cell dependence. The T-cell cytokines, IL-3, IL-4, IL-9, and IL-10, have all been shown to be involved in mast cell growth, and IL-3 injected into nude (athymic) mice results in MMC development (Abe et al., 1988). However, mutant mice with defects of the *c-kit* proto-oncogene (*W* locus on chromosome 5) have T-cells that produce IL-3 but lack proper mast cell development. In culture, IL-4 is synergistic to the actions of IL-3-dependent proliferation of mast cells, but does not alone maintain high levels of the cells. IL-4 also stimulates the production of B-cells and of importance perhaps to mast cells, IgE synthesis (Paul and Ohara, 1987). IL-9 was found to be identical with mast cell growth-enhancing activity (MEA). Although it does not itself enhance proliferation of BMCMC, it does increase IL-3-dependent proliferation. Similarly, IL-10 alone is not proliferative, but does give optimal growth in combination with IL-3 and IL-4. This trio of cytokines is characteristic of activated T-cells, and may be of greatest importance in certain immune reactions, especially parasitic infections.

### OTHER GRANULOCYTES

Granulopoiesis is the thymus has been extensively documented since the late 1800s (see Bhatal and Campbell, 1965). More recently, granulocyte development has been shown in rodents (Sin and Sainte-Marie 1965; von Haelst, 1967; Kendall and Blackett, 1983; Kendall et al., 1985; Boshnakova, 1990) humans (Downey, 1948; Bhatal and Campbell, 1965), cats (Pack and Chapman, 1980), and rabbits (Downey, 1948; Westermann and Engelbert, 1969a, 1969b, 1969c). The embryonic and fetal stages of many animals show development of granulocytes within the thymus, and this activity may continue into adult life. Westermann and Engelbert (1969a, 1969b) made a detailed study of the numbers and distribution of different granulocytes (eosinophils of two morphological forms, heterophils and basophils) at different life stages and under conditions of parasitic infection. The two forms of eosinophils were regarded as arising from different precursors in the thymus and it was found that their relative numbers varied with parasitic infections. Young rabbits had more eosinophils and fewer basophils than younger animals, and old animals and parasitized rabbits had the greatest concentrations of granulocytes. The use of thymectomized rats (Basten and Beeson, 1970) showed that the eosinophilia induced by *Nippostrongylus brasiliensis* was under T-lymphocyte control and this was confirmed in athymic rats (Ogilvie et al., 1980; Letonja et al., 1988). However, eosinophilia in the rat in response to *Fasciola hepatica* is not thymus-dependent (Doy and Hughes, 1982).

Immature and mature granulocytes are found in the cortex (often the outer cortex, where myelocytes can align themselves in rows under the capsule), and in the medulla. In young animals and under certain conditions in adults, such as induced anemia (Fig. 4a), development occurs in nests of eosinophils throughout the cortex (Kendall, 1978; Kendall and Blackett, 1983; Kendall et al., 1985). Granulocytes in the connective tissues of the septa and capsule are generally recorded as more mature, and may be cells entering or exiting the thymus. In many avian thymuses, mature eosinophils were found within vacuoles in or close to the necrotic center of Hassall's corpuscles (Kendall and Frazier, 1979). Associations of this nature were also observed in children (Bhatal and Campbell, 1965; Muller, 1977) and in rabbits (Westermann and Engelbert, 1969b). It has been suggested that they may be phagocytosing antigen-antibody complexes at this site because both antigen (Marshall and White, 1961) and immunoglobulins (Henry and Anderson, 1990) accumulate in Hassall's corpuscles.

### CONCLUSIONS

The thymus in the embryo and in most young animals including man can be extensively hemopoietic, possibly by the action of growth factors and cytokines creating permissive microenvironments. See Table 1. It is not surprising, therefore, that the thymus also can support hemopoiesis in adult life. Several important questions arise from this activity. Does an imbalance in the body's normal blood-cell composition favor hemopoiesis in the thymus, and/or do alterations to the thymic microenvironment predispose to thymic hemopoiesis? Do foci of hemopoiesis depend on activation of previously dormant multipotential stem cells or on the entry of stem cells from the circulation? To what extent are stem cells that may enter the adult thymus committed to develop into a specific lineage? However, now that it is clear that the thymus microenvironment is not restricted to T-cell development, the other hemopoietic activities will provide a rich research area where progress should be rapid, because many of the necessary tools are already available.

TABLE 1
The Presence of Non-T-Lineage Cells in the Thymus of Mammals

| Intrathymic cells | Embryo | Adult |
|---|---|---|
| Multipotential stem cells | Present | Probable |
| Immature B-cells | Present | Present |
| Immature erythroid cells | Present | Present |
| Immature mast cells | Not known | Present |
| Other immature granulocytes | Present | Present |

Because immature myeloid cells generally do not circulate in the blood of adults, except in disease, the presence of immature forms in the normal thymus is indicative of development and differentiation from multipotential or committed precursors. Whether these precursors lie dormant from embryonic days or enter the thymus from the circulation is not known.

### ACKNOWLEDGMENTS

I am grateful to the Volkswagen Stiftung and the Welton Foundation for funding the current work of the Thymus Laboratory at the Babraham Institute.

*(Received November 7, 1994)*

*(Accepted February 20, 1995)*

### REFERENCES

Abe T., Ochiai H., Minamishima Y., and Nawa Y. (1988). Induction of intestinal mastocytosis in nude mice by repeated injection of interleukin-3. Int. Arch. Allergy Appl. Immunol. **86:** 356–358.

Abou-Rabia N., and Kendall, M.D. (1994). Involution of the rat thymus in experimentally induced hypothyroidism. Cell Tissue Res. **277:** 447–455.

Adkins B., Tidmarsh G., and Weissman I.L. (1988). Normal thymic cortical epithelial cells developmentally regulate the expression of a B-lineage transformation-associated antigen. Immunogenetics **27:** 180–186.

Albert S., Wolf P., and Pryjma I. (1965a). Evidence of erythropoiesis in the thymus of mice. J. Retic. Soc. **2:** 30–39.

Albert S., Wolf P., Pryjma I., and Vazquez J. (1965b). Variations in morphology of erythroblasts of normal mouse thymus. J. Retic. Soc. **2:** 158–171.

Albert S., Wolf P., Pryjma I. and Vazquez J. (1966). Erythropoiesis in the human thymus. Amer. J. Clin. Pathol. **45:** 460–464.

Aldenborg F., and Enerback L. (1985). Thymus dependence of connective tissue mast cells: A quantitative cytofluorometric study of the growth of peritoneal mast cells in normal and athymic rats. Int. Arch. Allergy Appl. Immunol. **78:** 277–282.

Andreu-Sanchez J.L., Faro J., Alonso J.M., Paige C.J., Martinez A., and Marcos M. (1990). Ontogenetic characterization of thymic B lymphocytes. Analysis in different mouse strains. Eur. J. Immunol. **20:** 1767–1773.

Anonymous (1959). Thymoma and red cell aplasia. Brit. Med. J. May 2: 1174.

Bacchus S., and Kendall M.D. (1975). Histological changes with enlargement and regression of the thymus glands of the red-billed quelea *Quelea quelea* L. (Ploceidiae: weaverbirds). Philos. Trans. Roy. Soc. B **273:** 65–78.

Basten A., and Beeson P.B. (1970). Mechanisms of eosinophilia II. Role of the lymphocyte. J. Exp. Med. **131:** 1288–1305.

Befus A.D., Johnston N., Nielsen L., Bienenstock J., Butler J., and Cosmos E. (1981). Thymic mast cell deficiency in avian muscular dystrophy. Thymus **3:** 369–376.

Benner R., Meima F., van der Meulen G.M., and van Ewijk W. (1974). Antibody formation in mouse bone marrow. III. Effects of route of priming and antigen dose. Immunology **27:** 747–760.

Bhatal P.S., and Campbell P.E. (1965). Eosinophil leukocytes in the child's thymus. Austral. Ann. Med. **14:** 210–213.

Borgeois N., Bergmans G., and Buyssens N. (1981). The thymus as haemopoietic tissue of non lymphoid cells. Virchows Arch. **391:** 81–89.

Boshnakova E. (1990). Eosinophilic granulocytes in the mouse thymus. Eksper. Med. Morphol. **29:** 49–51. (In Bulgarian, not seen).

Burnet F.M. (1965). Mast cells in the thymus of NZB mice. J. Pathol. Bacteriol. **89:** 271–284.

Clarke A.G., and Kendall M.D. (1989). Histological changes in the thymus during mouse pregnancy. Thymus **14:** 65–78.

Downey H. (1948). Cytology of rabbit thymus and regeneration of its thymocytes after irradiation; with some notes on the human thymus. Blood **3:** 1315–1341.

Doy T.G., and Hughes D.L. (1982). The role of the thymus in eosinophil response of rats infected with Fasciola hepatica. Clin. Exp. Immunol. **47:** 74–76.

Ezine S., Weissman I.L., and Rouse R.V. (1984). Bone marrow cells give rise to distinct cell clones within the thymus. Nature **309:** 629–631.

Farinas M.C., Adkins B., Stall A.M., Weissman I.L., and Strober S. (1990). B cell infiltration of the thymic medulla in New Zealand black, New Zealand white, and (New Zealand black-New Zealand white)$F_1$ mice. Arth. Rheumat. **33:** 702–710.

Fend F., Nachbaur D., Oberwasserlechner F., Kreczy A., Huber H., and Müller-Hermelink H.K. (1991). Phenotype and topography of human thymic B cells. An immunohistologic study. Virchows Arch. B Cell Pathol. **60:** 381–388.

Frazier J.A. (1973). Ultrastructure of the chicken thymus. Zeitschrift für Zellforschung **136:** 191–205.

Frey J.R., Ernst B., Surh C.D., and Sprent J. (1992). Thymus-grafted SCID mice show transient thymopoiesis and limited depletion of $V\beta11^+$ T cells. J. Exp. Med. 175: 1067–1071.

Fronfria J., Barrutia M.G., Garrido E., Ardavin C.F., Villena A., and Zapata A. (1983). Erythropoiesis in the thymus of the spotless starling *Sturnus unicolor*. Cell Tissue Res. **232:** 445–455.

Fujii N., Itoyama Y., Tebira T., and Kuroiwa T. (1983). Subsets of lymphoid cells in blood and thymus in myasthenia gravis. Monoclonal antibody analysis. J. Neuroimmunol. **4:** 151–159.

Galy A.H.M., and Spitz H. (1991). IL-1, IL-4, and IFN-γ differentially regulate cytokine production and cell surface molecule expression in cultured human thymic epithelial cells. J. Immunol. **147:** 3823–3830.

Ginsburg H., and Sachs L. (1963). Formation of pure suspensions of mast cells in tissue culture by differentiation of lymphoid cells from the mouse thymus. J. Nat. Cancer Inst. **31:** 1–40.

Henry L., and Anderson G. (1990). Immunoglobulins in Hassall's corpuscles of the human thymus. J. Anat. **168:** 185–197.

Hofmann W.J., Momburg F., and Moller P. (1988). Thymic medullary cells expressing B lymphocyte antigens. Human Pathol. **19:** 1280–1287.

Huang E., Nocka K., Beier D.R., Chu T-Y., Buck J., Lahm H.-W., Wellner D., Leder P., and Besmer P. (1990). The hemopoietic growth factor KL is encoded by the *Sl* locus and is the ligand of the *c-kit* receptor, the gene product of the *W* locus. Cell **61:** 953–963.

Huang E.J., Nocka K.H., Buck J., and Besmer P. (1992). Differential expression and processing of two cell associated forms of the *c-kit* ligand: KL-1 and KL-2. Mol. Biol. Cell **3:** 349–362.

Ikuta K., Kina T., MacNeil I., Uchida N., Peault B., Chien Y.H., and Weissman I.L. (1990). A developmental switch in thymic lymphocyte maturation potential occurs at the level of hemopoietic stem cells. Cell **62:** 863–874.

Inaba M., Inaba K., Adechi Y., Nango K.-I., Ogata H., Muramatsu S., and Ikehara S. (1990). Functional analysis of thymic $CD5^+$ B cells. J. Exp. Med. **171:** 321–326.

Inaba M., Inaba K., Hosono M., Kumamoto T., Ishida T., Muramatsu S., Masuda T., and Ikehara S. (1991). Distinct mechanisms of neonatal tolerance induced by dendritic cells and thymic B cells. J. Exp. Med. **173:** 549–559.

Isaacson P.G., Norton A.J., and Addis B.J. (1987). The human thymus contains a novel population of B lymphocytes. Lancet **2(8574):** 1488–1491.

Ishizaka T., Okudaira H., Mauser L.E., and Ishizaka K. (1976). Development of rat mast cells in vitro. I. Differentiation of mast cells from thymus cells. J. Immunol. **116:** 747–754.

Jacobs E.M., Hutter R.V.P., Pool J.L., and Ley A.B. (1959). Benign thymoma and selective erythroid aplasia of the bone-marrow. Cancer **12:** 47–57.

Karagogeos D., and Wortis H.H. (1987). Thymus graft induced B cell development in nude, X-linked immune deficient mice. Eur. J. Immunol. **17:** 141–144.

Kawanishi H., Medicus R.G., and Palaszynski E.W. (1986). Mast cells induced by interleukin 3 from native murine thymus cells. Scand. J. Immunol. **24:** 29–38.

Kelemen E., Calvo W., and Fliedner T.M. (1979). Atlas of human hemopoietic development (Berlin: Springer-Verlag).

Kendall M.D. (1975a). EMMA-4 analysis of iron in cells of the thymic cortex of a weaver bird (*Quelea quelea*). Philos. Trans. Roy. Soc. Part B **273:** 79–82.

Kendall M.D. (1975b). Sizes and numbers of nuclei in the cortex of thymus glands of red-billed weavers (*Quelea quelea*). Cell Tissue Res. **164:** 233–249.

Kendall M.D. (1978). The effect of haemorrhage on the cell populations of the thymus and bone marrow in wild starlings (*Sturnus vulgaris*). Cell Tissue Res. **190:** 459–479.

Kendall M.D. (1979). Ultrastructural studies on erythropoiesis in the avian thymus II. A stereological analysis of the lymphoid and erythroid cells. Cell Tissue Res. **199:** 63–74.

Kendall M.D. (1980a). Avian thymus glands: A review. Dev. Comp. Immunol. **4:** 191–210.

Kendall M.D. (1980b). The occurrence of erythropoiesis in the thymus glands of the bank vole *Clethrionomys glareolus*. Cell Tissue Res. **212:** 307–314.

Kendall M.D. (1981). The thymus and haemopoiesis. *In:* Advances in morphology of cells and tissues, XIth International Congress of Anatomy B, pp. 221–230.

Kendall M.D., and Blackett N.M. (1983). Ultrastructural studies on the thymus gland after the administration of phenylhydrazine to bank voles (*Clethrionomys glareolus*). Cell Tissue Res. 232: 201–219.

Kendall M.D., and Frazier J.A. (1979). Ultrastructural studies on erythropoiesis in the avian thymus. I. Description of cell types. Cell Tissue Res. **199:** 37–61.

Kendall M.D., Jaffe P., and Yoffey J.M. (1985). The mouse thymus in hypoxia and rebound: A histological study. J. Anat. **142:** 85–102.

Kendall M.D., and Singh J. (1980). The presence of erythroid cells in the thymus gland of man. J. Anat. **130:** 183–189.

Kendall M.D., and Ward P. (1974). Erythropoiesis in an avian thymus. Nature **249:** 366–367.

Kendall M.D., and Warley A. (1986). The elemental content of mast cell granules measured by X-ray microanalysis of rat thymic tissue sections. J. Cell Sci. **83:** 77–87.
Kimoto H., Shirasawa T., Taniguchi M., and Takemori T. (1989). B cell precursors are present in the thymus during early development. Eur. J. Immunol. **19:** 97–104.
Kitamura Y., Yokoyama M., Matsuda H., Ohno T., and Mori K.J. (1981). Spleen colony forming cell as common precursor for tissue mast cells and granulocytes. Nature **291:** 159–160.
Krantz S.B. (1990). Pure red cell aplasia. *In:* Surgery of the thymus, Givel J.-C. (Berlin: Springer Verlag), pp. 181–188.
Kupper H., Ziermann S., Feibig H., Vogt S., and Heidrich L. (1989). Secondary follicles in the thymus. Immunohistologic characterization of B lymphocytes using monoclonal antibodies. Zent. All. Pathol. Pathol. Anat. **135:** 269–275.
Leprince C., Cohen-Kaminsky S., Berrih-Aknin S., Garabedian B.V., Treton D., Galanaud, P., and Richard Y. (1990). Thymic B cells from myasthenia gravis patients are activated B cells. J. Immunol. **45:** 2115–2122.
Letonja T., Hammerberg C., Davis S., and Hammerberg B. (1988). Taenia taeniaformis: Cellular reconstruction of athymic mice and role of L3T4$^+$ helper T lymphocytes in early infection. J. Parasitol. **74:** 985–992.
Marshall A.H.E., and White R.G. (1961). The immunological reactivity of the thymus. Brit. J. Pathol. **42:** 379–385.
Matsui Y., Toksoz D., Nishikawa S., Nishikawa S., Williams D., Zsebo K., and Hogan B.L. (1991). Effects of Steel factor and leukemia inhibitory factor on murine primordial germ cells in culture. Nature **353:** 750–752.
Mayrhofer G., and Fisher R. (1979). Mast cells in severely T-cell depleted rats and the response to infestation with *Nippostrongylus brasiliensis.* Immunology **37:** 145–155.
Maximov A. (1909). Untersuchungen über Blut und Bindegewebe. II. Ueber die Histogenese der Thymus bei Saugetieren. Archiv Mikro. Anat. **74:** 525–621.
Mazda O., Watanabe Y., Gyotoku J.-I. and Katsura Y. (1991). Requirement of dendritic cells and B cells in the clonal deletion of Mls-reactive T cells. J. Exp. Med. **173:** 539–547.
Michie A.A. and Rouse R.V. (1991). Traffic of peripheral B and T lymphocytes to hyperplastic, preneoplastic thymuses of AKR mice. Amer. J. Pathol. **138:** 1015–1025.
Middleton G. (1967). The incidence of follicular structures in the human thymus at autopsy. Austral. J. Exp. Biol. Med. Sc. **45:** 189–199.
Moore N.C., Anderson G., Smith C.A., Owen J.J.T., and Jenkinson E.J. (1993). Analysis of cytokine gene expression in subpopulations of freshly isolated thymocytes and thymic stromal cells using semiquantitative polymerase chain reaction. Eur. J. Immunol. **23:** 922–927.
Muller E. (1977). Localization of eosinophils in the thymus by the peroxidase reaction. Histochemistry **52:** 273–279.
Nakano T., Sonoda T., Hayashi C., Yamatodami A., Kanayama Y., Asai H., Yonezawa T., Kitamura Y., and Gali S.J. (1985). Fate of bone marrow-derived cultured mast cells after intracutaneous, interperitoneal, and intravenous transfer into genetically mast cell deficient $W/W^v$ mice: Evidence that cultured mast cells can give rise to both "connective tissue-type" and "mucosal" mast cells. J. Exp. Med. **162:** 1025–1043.
Nango K.-I., Inaba M., Inaba K., Adachi Y., Than S., Ishida T., Kuomoto T., Uyama M., and Ikehara S. (1991). Ontogeny of thymic B cells in normal mice. Cell. Immunol. **133**(1): 109–115.
Ogilvie B.M., Askenase P.W., and Rose M.E. (1980). Basophils and eosinophils in three strains of rats in athymic (nude) rats following infection with the nematodes *Nippostrongylus brasiliensis* or *Trichinella spiralis.* Immunology **39:** 385–389.
Pabst R.R., Binns R.M., and Westermánn J. (1989). What is the function of peripheral lymphocytes migrating to the thymus and of B lymphocytes proliferating in the thymus? Thymus **13:** 149–156.
Pack F.D., and Chapman W.L. (1989). Light and electron microscopic evaluation of thymuses from feline leukemia virus-infected kittens. Exp. Pathol. **18:** 96–110.
Parry H.E.O., Kilpatrick G.S., and Hardisty R.M. (1959) Red cell aplasia and benign thymoma. Studies on a case responding to prednisolone. Brit. Med. J. May 2: 1154–1156.
Paul W.E., and Ohara J. (1987). B-cell stimulatory factor-1/ interleukin 4. Annual Rev. Immunol. **5:** 429–459.
Peault B., Khazaal I., and Weissman I.L. (1994). In vitro development of B cells and macrophages from early mouse fetal thymocytes. Eur. J. Immunol. **24:** 781–784.
Rosai J., and Levine G.D. (1976). Tumors of the thymus. *In:* Atlas of tumor pathology, 2d series, Fascicle 13 (Washington, DC: Armed Forces Institute of Pathology), pp. 26–33.
Ross J.F., Finch S.C., Street R.B. Jr, and Streider J.W. (1954). The simultaneous occurrence of benign thymoma and refractory anaemia. Blood **9:** 935–952.
Ruitenberg E.J. and Elgesma A. (1976). Absence of intestinal mast cell response in congenitally athymic mice during *Trichinella spiralis* infection. Nature **264:** 258–260.
Safar D., Berrih-Aknin S., and Morel E. (1987). In vitro anti-acetylcholine receptor antibody synthesis by myasthenia gravis patient lymphocytes correlated with thymic histology and thymic epithelial-cell interactions. J. Clin. Immunol. **7:** 225–234.
Sharkis S.J., Cremo C., Collector M.I., Noga S.J., and Donnenberg A.D. (1986). Thymic regulation of hemopoiesis. III. Isolation of helper and suppressor populations using counterflow centrifugal elutriation. Blood **68:** 787–789.
Sin Y.M., and Sainte-Marie G. (1965). Granulocytopoiesis in the rat thymus. I. Description of the cells of the neutrophilic and eosinophilic series. Brit. J. Haematol. **11:** 613–623.
Spangrude G.J., Heimfeld S., and Weissman I.L. (1988). Purification and characterization of mouse hematopoietic stem cells. Science **241:** 58–62.
Spangrude G.J., and Scollay R. (1990). A simplified method for enrichment of mouse hematopoietic stem cells. Exp. Hematol. **18:** 920–926.
Spencer J., Choy M., Hussell T., Papadaki L., Kington J.P., and Isaacson P.G. (1992). Properties of human thymic B cells. Immunology **75:** 596–600.
Sprent J. (1973). Circulating T and B lymphocytes of the mouse. I. Migratory properties. Cell. Immunol. **7:** 10–39.
Sprent J., and Bruce J. (1984). Physiology of B cells in mice with X-linked immunodeficiency. II. Influence of the thymus and mature T cells on B cell differentiation. J. Exp. Med. **160:** 335–340.
Taylor C.R., and Skinner J.M. (1976). Evidence for significant hemopoiesis in the human thymus. Blood **47:** 305–313.
Testi R., D'Ambrosio D., De Maria R., and Santoni A. (1994). The CD69 receptor: A multipurpose cell-surface trigger for hematopoietic cells. Immunol. Today **15:** 479–483.
Than S., Inaba M., Inaba K., Fukaba Y., Adachi Y., and Ikehara S. (1992). Origin of thymic and peritoneal Ly-1 B cells. Eur. J. Immunol. **22:** 1299–1303.
Tsunoda J.-I., Okada S., Suda J., Nagoyoshi K., Nakanchi H., Hatake K., Miura Y., and Suda T. (1991). In vivo stem cell function of interleukin-3-induced blast cells. Blood **78:** 318–322.
Vandekerckhove B.A.E., Jones D., Punnonen J., Schols D., Lin H-C.L., Duncan B., Bacchetta R., de Vries J.E., and Roncarolo M.-G. (1993). Human Ig production and isotype switching in severe combined immunodeficient-human mice. J. Immunol. **151:** 128–137.
Vetters J.M., and Barclay R.S. (1973). The incidence of germinal centers in thymus glands of patients with congenital heart disease. J. Clin. Pathol. **26:** 583–591.
Vincent A., Thomas H.C., Scadding G.K., and Newson-Davis J. (1978). In vitro synthesis of anti-acetylcholine-receptor antibodies by thymic lymphocytes in myasthenia gravis. Lancet **1:** 305–307.

von Haelst U. (1967). Light and electron microscopic study of the normal and pathological thymus of the rat. I. The normal thymus. Z Zellforsch. **77:** 534–553.

Ward P., and Kendall M.D. (1975). Morphological changes in the thymus of young and adult red-billed queleas, *Quelea quelea* (Aves). Philos. Trans. Roy. Soc. Part B **273:** 55–64.

Westermann J.E.M., and Engelbert V.E. (1969a). The distribution of eosinophilic and heterophilic granulocytes in the rabbit thymus. Canad. J. Zool. **47:** 89–94.

Westermann J.E.M., and Engelbert V.E. (1969b). Numbers of granulocytes in thymic imprints of rabbits of different ages and degrees of coccidial parasitism. Canad. J. Zool. **47:** 1355–1362.

Westermann J.E.M., and Engelbert V.E. (1969c). The development of eosinophilic granulocytes in the rabbit thymus. Canad. J. Zool. **47:** 1381–1387.

Whitlock C.A., Tidmarsh G.F., Muller-Sieburg C., and Weissman I.L. (1987). Bone marrow stromal cell lines with lymphopoietic activity express high levels of a pre-B neoplasia-associated molecule. Cell **48:** 1009–1021.

Wight P.A.L. (1970). The mast cells of *Gallus domesticus.* I. Distribution and ultrastructure. Acta Anat. **75:** 100–113.

Williams C.L., and Lennon V.A. (1986). Thymic B lymphocyte clones from patients with myasthenia gravis secrete monoclonal striational autoantibodies reacting with myosin, αactinin or actin. J. Exp. Med. **164:** 1043–1059.

Wirt D.P,, Grogan T.M., Nagle R.B., Copeland J.G., Richter L.C., Rangel. C.S., Schuchardt M., Fosse J., and Layton J.M. (1988). A comprehensive immunotopographical map of human thymus. J. Histochem. Cytochem. **36:** 1–12.

Zoller M. (1990). Intrathymic presentation of nominal antigen by B cells. Int. Immunol. **2:** 427–434.

Zsebo K.M., Williams D.A., Geissler E.N., Brondy V.C., Martin F.H., Atkins H.L., Hsu R.-Y., Birkett N.C., Okino K.H., Murdock D.C., Jacobsen F.W., Langley K.E., Smith K.A., Takeishi T., Cattanach B.M., Galli S.J., and Suggs S.V. (1990). Stem cell factor is encoded at the *SI* locus of the mouse and is the ligand for *c-kit* tyrosine kinase receptor. Cell **63:** 213–224.

